# *In Silico* Investigation of Potential Src Kinase Ligands from Traditional Chinese Medicine

**DOI:** 10.1371/journal.pone.0033728

**Published:** 2012-03-21

**Authors:** Weng Ieong Tou, Calvin Yu-Chian Chen

**Affiliations:** 1 School of Medicine, China Medical University, Taichung, Taiwan; 2 Laboratory of Computational and Systems Biology, China Medical University, Taichung, Taiwan; 3 Department of Medical Research, China Medical University Hospital, Taichung, Taiwan; 4 Department of Biotechnology, Asia University, Taichung, Taiwan; 5 Department of Biomedical Informatics, Asia University, Taichung, Taiwan; 6 China Medical University Beigang Hospital, Yunlin, Taiwan; Spanish National Cancer Center, Spain

## Abstract

Src kinase is an attractive target for drug development based on its established relationship with cancer and possible link to hypertension. The suitability of traditional Chinese medicine (TCM) compounds as potential drug ligands for further biological evaluation was investigated using structure-based, ligand-based, and molecular dynamics (MD) analysis. Isopraeroside IV, 9alpha-hydroxyfraxinellone-9-O-beta-D-glucoside (9HFG) and aurantiamide were the top three TCM candidates identified from docking. Hydrogen bonds and hydrophobic interactions were the primary forces governing docking stability. Their stability with Src kinase under a dynamic state was further validated through MD and torsion angle analysis. Complexes formed by TCM candidates have lower total energy estimates than the control Sacaratinib. Four quantitative-structural activity relationship (QSAR) *in silico* verifications consistently suggested that the TCM candidates have bioactive properties. Docking conformations of 9HFG and aurantiamide in the Src kinase ATP binding site suggest potential inhibitor-like characteristics, including competitive binding at the ATP binding site (Lys295) and stabilization of the catalytic cleft integrity. The TCM candidates have significantly lower ligand internal energies and are estimated to form more stable complexes with Src kinase than Saracatinib. Structure-based and ligand-based analysis support the drug-like potential of 9HFG and aurantiamide and binding mechanisms reveal the tendency of these two candidates to compete for the ATP binding site.

## Introduction

Src kinases are nonreceptor tyrosine kinases that are of physiological importance in cell survival, bone metabolism, angiogenesis, proliferation, migration, and invasion [1]. Overexpression of Src kinase has been linked to various cancers and is now a well-established proto-oncogene [2]–[7]. The physiological pathway involved in hypertension is also associated with Src-dependent signaling pathways, suggesting a potential link between hypertension and Src [8]–[15].

**Figure 1:**
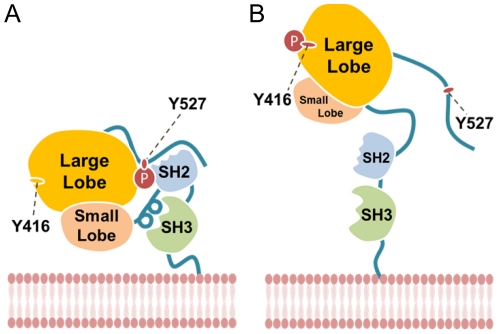
Components of Src and its activation mechanisms. (A) SH2 binds to the phosphorylated Tyr530 and SH3 binds with prolines on the linker domain, effectively locking the Src in an inactive closed conformation. (B) Src is activated when Tyr530 is dephosphorylated and Tyr416 within the catalytic domain is autophosphorylated.

Figure 1 illustrates the components of Src kinase and its activation mechanism [16], [17]. In general terms, the catalytic activity of Src is co-regulated by SH3 and SH2 domains. Src is locked in the closed conformation (inactive) when SH2 binds to the phosphorylated Tyr530, and SH3 binds with prolines on the linker domain (Figure 1A). When Tyr530 is dephosphorylated, Src assumes an open conformation, achieving full activity when Tyr416 within the catalytic domain is autophosphorylated (Figure 1B). This opening of the Src structure frees the SH2 and SH3 domains to interact with surface receptors such as focal adhesion FAK and initiate downstream signaling governing the aforementioned physiological pathways [1]. In this regard, inactivation of Src can be achieved through hindering disassembly of the regulatory SH2 and SH3 Src domains, or by inhibiting ATP binding to the Src catalytic site [16], [18].

**Figure 2:**
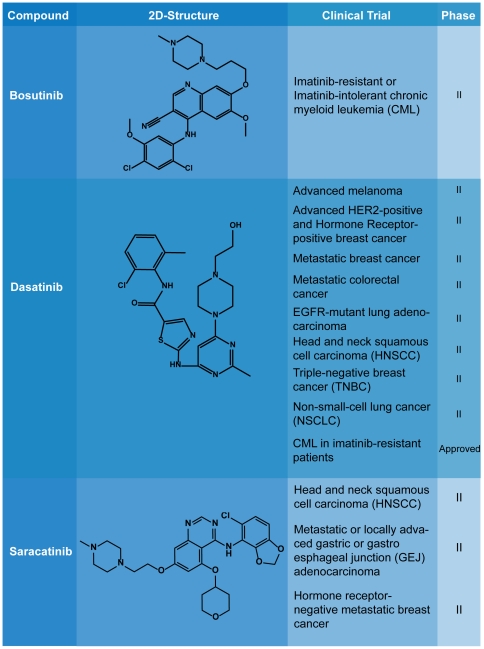
Scaffolds of Bosutinib, Dasatinib, amd Saracatinib, and their respective status in clinical trials.

Many small molecular Src inhibitors have been identified due to the involvement of Src in cancer. Comprehensive reviews on such advancements are detailed elsewhere [16]. Most Src inhibitors discovered to date are Type I inhibitors that compete with ATP for binding at the ATP binding pocket [19], [20]. Structures of the three most studied Type I inhibitors Bosutinib, Dasatinib, and Saracatinib and are shown in Figure 2 along with their respective status in clinical trials [21]–[32]. The varying efficacies of these commercial drugs highlight the need for novel compounds that can exhibit more consistent inhibition of Src.

The goal of this research is to investigate novel small compounds from traditional Chinese medicine (TCM) that may be potential Src kinase ligands. During the past decade, our laboratory has focused on constructing the most comprehensive TCM database (TCM Database@Taiwan) (http://tcm.cmu.edu.tw/) [33]. In addition, we've also developed the first cloud-computing webserver based on TCM Database@Taiwan (http://iscreen.cmu.edu.tw/) [34] and an integrative website combining TCM and systems biology (http://iSMART.cmu.edu.tw/) [35]. Utilizing these TCM computational resources, several novel lead compounds from TCM with application potential for different diseases have been successfully uncovered [36]–[44]. In the current research, we utilize the newly updated TCM Database@Taiwan to screen for novel, TCM-origin ligands with drug-like properties against Src kinase.

## Results and Discussion

### Docking

**Figure 3:**
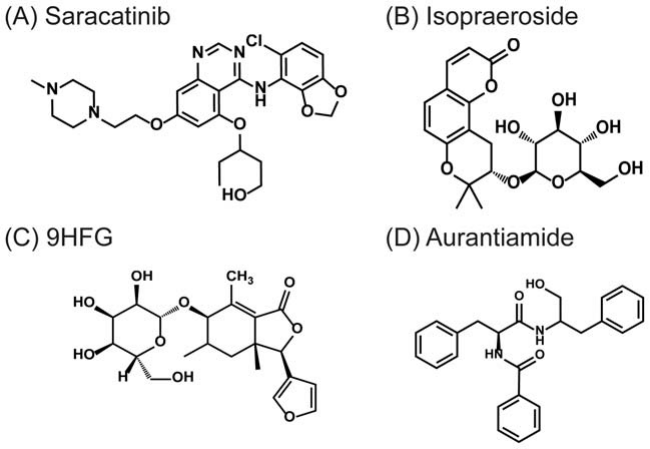
Structural scaffolds of TCM candidates and Saracatinib. (A) Saracatinib, (B) Isopraeroside IV, (C) 9HFG, and (D) aurantiamide.

Based on the DockScore, top ranking TCM candidates selected for further investigation were Isopraeroside IV, 9alpha-hydroxyfraxinellone-9-O-beta-D-glucoside (9HFG), and aurantiamide (Table 1). Isopraeroside IV is a coumarin isolated from the root of *Angelica dahurica* [45]. 9HFG originates from the root of *Dictamnus dasycarpus*, which has been used as a folk remedy for inflammation and skin ailments [46]. Aurantuamide is a composition of *Curcuma wenyujin*, a dried rhizome used for bleeding and menstrual disorders in traditional Chinese medicine. Structural comparisons of the TCM candidates with Saracatinib are illustrated in Figure 3.

**Table 1:** DockScore and related attributes of Sacracatinib and top three TCM candidates calculated by Discovery Studio 2.5 (D.S. 2.5).

Name	Ligand Internal Energy	Binding Energy	Dock Score
Isopraeroside IV	−42.52	−63.39	97.28
9HFG	−35.33	−60.24	93.12
Aurantiamide	−37.24	−57.12	83.02
Saracatinib*	34.20	−60.81	27.09

Candidates ranked by Dock Score.

* Control.

9HFG: 9alpha-hydroxyfraxinellone-9-O-beta-D-glucoside.

DockScore is the negative sum of the ligand/receptor interaction and the ligand internal energy calculated by DS. For clarification purposes, the ligand internal energy consists of a van der Waals (vdW) term computed using a standard 9-6 (unsoftened) potential and an optional electrostatic term. Since TCM candidates and Saracatinib have similar predicted binding energies, differences in DockScore were primarily affected by the respective calculated ligand internal energies of each compound (Table 1). The low DockScore observed for Saracatinib is mainly due to its high calculated ligand internal energy. The positive ligand internal energy value is indicative of a highly strained chemical structure, a phenomenon commonly observed among synthetic compounds. By contrast, low calculated ligand internal energies of the TCM compounds suggest energetically stable structures in which intermolecular atoms are not too close to exhibit replusion and result in high DockScore values. Based on DS2.5 calculations, the TCM candidates have higher DockScores and binding energies comparable to Saracatinib, which may lead to equal if not better binding to the Src kinase binding site compared to Saracatinib.

**Figure 4:**
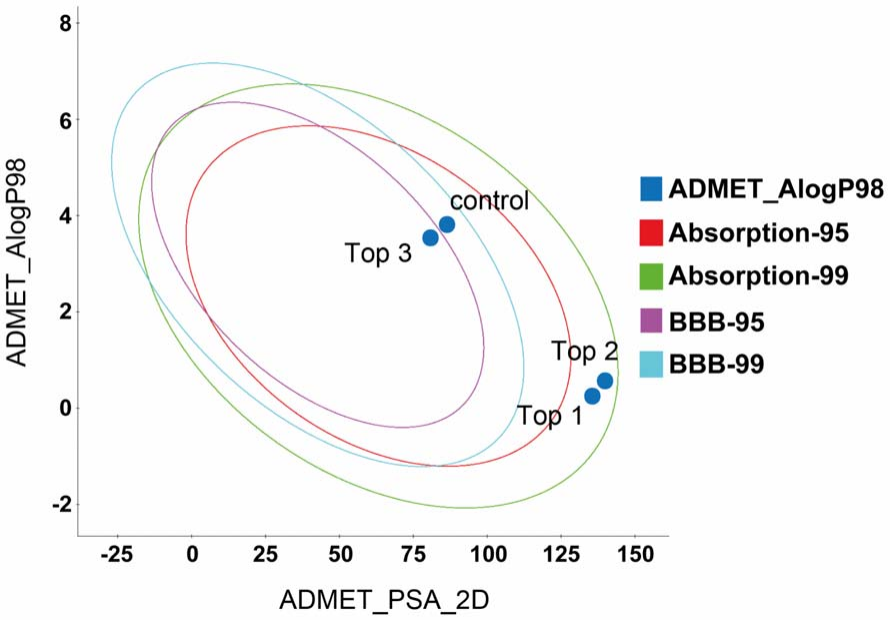
ADMET adsorption model of Saracatinib and the TCM candidates generated by DS 2.5.

The ADMET properties of the TCM candidates are summarized in Figure 4 and Table 2. All candidates were estimated to be within the 99% adsorption ellipse (Figure 4) and have good to moderate adsorption (Table 2). The TCM candidates are more highly bound to plasma proteins, and less likely to inhibit cytochrome P4502D6 metabolism.

**Table 2:** Adsorption, distribution, metabolism, toxicity properties and predicted pIC_50_ of Saracatinib and the top three TCM compounds.

Name	ADMET Adsorption Level^1^	PPBLevel^2^	CYP2D6Probability^3^	Hepatoxicity^4^	Bioactivity Prediction (pIC_50_)	
					SVM	MLR
Isopraeroside IV	1	2	0.356	0	6.0277	7.7282
9HFG	1	2	0.297	0	6.8590	7.5342
Aurantiamide	0	2	0.475	0	4.6490	4.4080
Saracatinib*	0	0	0.297	0	7.3036	4.9410

* Control.

1 ADMET Adsorption levels: 0 = Good; 1 = Moderate; 2 = Low; 3 = Very low.

2 Plasma Protein Binding: 0 = Binding <90% ;1 = Binding >90%; 2 = Binding >95%.

3 Inhibition probability of Cytochrome P450 2D6 enzyme.

4 Hepatoxicity: 0 = Nontoxic; 1 = Toxic.

**Figure 5:**
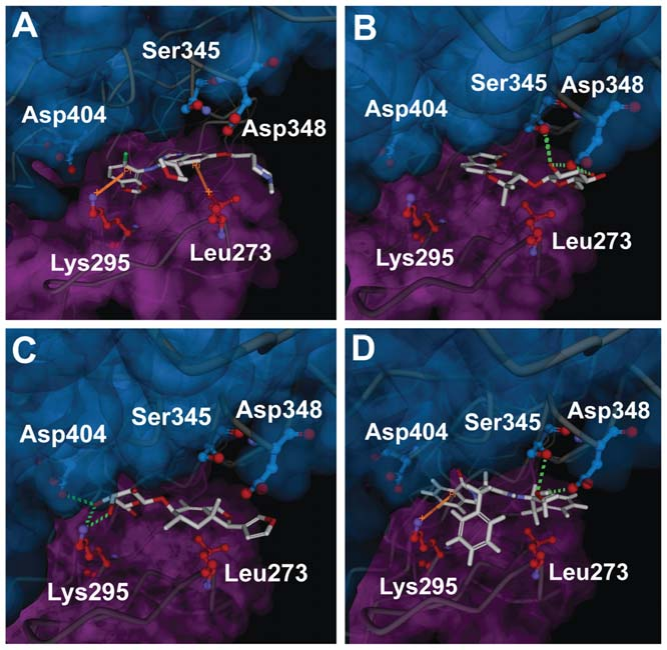
Docking poses of different ligands in Src kinase ATP binding pocket. Shown are snapshots of (A) Saracatinib,(B) Isopraeroside IV, (C) 9HFG, and (D) aurantiamide during docking simulation with Src kinase. Purple and blue surfaces are added to denote the small lobe (267–337) and large lobe (340–520) of Src kinase, respectively. Hydrogen bonds are shown as dotted green lines and pi-interactions are shown in orange. (A) Pi-interactions with Lys273 and Lys295 are critical for Saracatinib. (B) Isopraeroside IV docks to the outer region of the ATP binding pocket via H-bonds at Ser345 and Asp348. (C) 9HFG structure enables docking in the inner regions of the ATP binding pocket, forming H-bonds with Lys295 and Asp404. (D) Similar to Saracatinib, aurantiamide forms pi-interactions with Lys295, in addition to H-bonds with Ser345 and Asp348.

As our designated binding site overlapped with the Src kinase ATP binding pocket, it is referred to as the latter for clarification purposes. Docking poses of the candidates in the ATP binding pocket are illustrated in Figure 5. The docking model of Saracatinib indicates π-interactions with Leu273 and Lys295, and an H-bond with Met341 within the ATP binding pocket (Figure 5A). Isopraeroside IV formed a total of five H-bonds with Met341, Ser345, Asp348 (Figure 5B), possibly contributing to the high estimated binding energy in Table 1. The three hydroxyl groups on the cyclohexane moiety of 9HFG interacted with Lys295, Met341, and Asp404, forming a total of five H-bonds (Figure 5C) in which three were formed with Lys295. Auraniamide interacted with Leu273, Lys295, Ser345, Asp348, forming two π interactions at the terminal benzenes with Leu273 and Lys295 and H-bonds with Ser345 and Asp 348 (Figure 5D).

**Figure 6:**
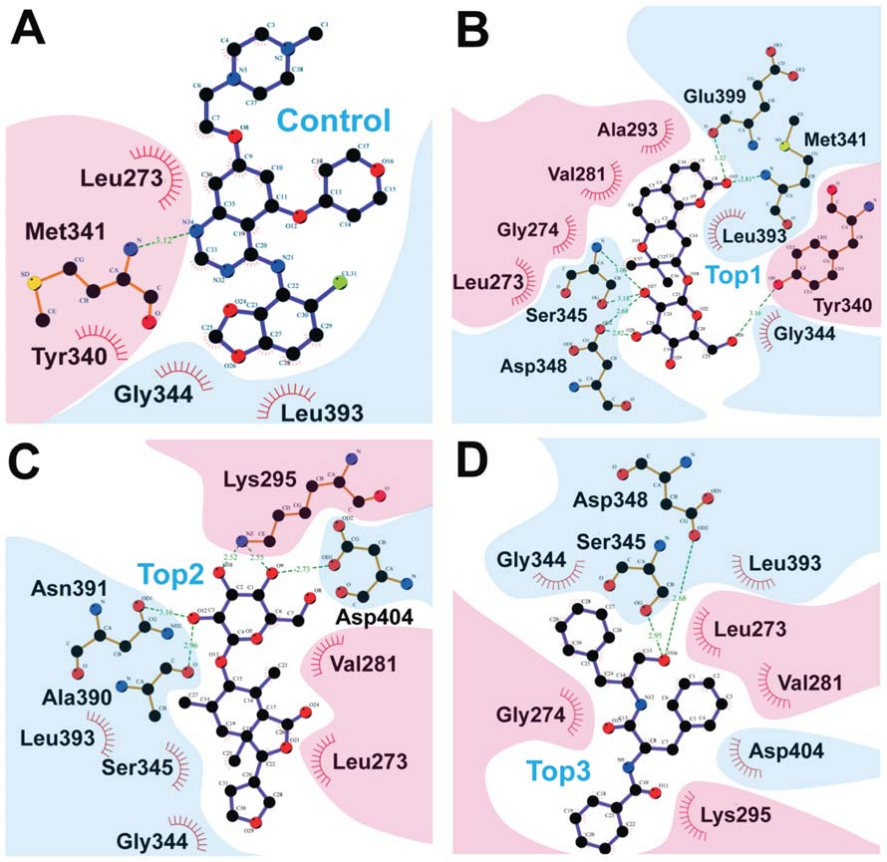
Ligplot diagrams illustrating protein-ligand interactions during docking. (A) Saracatinib, (B) Isopraeroside IV, (C) 9HFG, and (D) aurantiamide. Hydrophobic interactions are represented by red spokes radiating towards the ligand atoms they contact. Ligands are represented in purple. C, N, O, and Cl atoms are represented in black, blue, red, and green, respectively. Pink backgrounds highlight amino acids of the small lobe (267–337), and blue background highlight those belonging to the large lobe (340–520). Hydrophobic interactions shown in this illustration are calculated through the Ligplot algorithm.

Ligplot diagrams illustrating hydrophobic and H-bond interactions are shown in Figure 6. In general, the higher ability of ligands to form hydrophobic interactions with hydrophobic amino acids of the binding site, the higher the binding affinity. As shown in Figure 6, TCM candidates are surrounded by hydrophobic amino acids, therefore are harder to remove from the ATP binding pocket under physiologically dynamic conditions. Less hydrophobic interactions were formed by Saracatinib. Minor variations in H-bond formation were observed in LigPlot diagrams compared to 3D-docking poses generated by DS 2.5 (Figure 5). Additional H-bonds were formed with Tyr340 and Glu399 by Isopraeroside IV (Figure 6B). 9HFG formed two additional H-bonds at Ala390 and Asn391 (Figure 6C). In auraniamide (Figure 6D), the H-bonds at Leu273 and Lys295 were replaced by hydrophobic interactions. These differences may be due to the DS cutoff distance of 2.5 Å or to the HBPLUS program used to calculate H-bonds in LigPlot. The number of H-bonds and hydrophobic interactions determined through LigPlot is summarized in Table 3. These simulation results suggest that TCM candidates have a higher number of stabilizing interactions than Saracatinib.

**Table 3:** Ligand-protein interactions determined through LigPlot.

Amino acid in Src kinase	Hydrophobic interactions^1^	Ligand			
		Saracatinib	Isopraeroside IV	9HFG	Aurantiamide
Leu273/Leu393	**+**	A/A	A/A	A/A	A/A
Gly274/Gly344	**+**	-/A	A/A	-/A	A/A
Val281	**+**	-	A	A	A
Ala293/Ala390	**+**	-	A/-	H1	-
Lys295	**+**	-	-	H2	A
Tyr340	**+**	A	H1	-	-
Met341	−	H1	H1	-	-
Ser345	−	-	H2	A	H1
Asp348/Asp404	−	-	H2/-	-/H1	H1/A
Asn391	−	-	-	H1	-
Glu399	−	-	H1	H1	-

1 Amino acid residues where hydrophobic interactions are observed are denoted by the “+” symbol.

A: hydrophobic interaction.

H1: form one hydrogen bond between ligand and amino acid.

H2: form two hydrogen bond between ligand and amino acid.

Key amino acids and important structural moieties can be summarized based on the docking simulation results. Leu273 is important for H-bonds and π-interactions, and ligands form interactions with its nonpolar, aliphatic alkyl group. Saracatinib, 9HFG, and auraniamide interact with Src kinase through Lys295. The importance of Lys295 is reinforced by the three H-bonds and two π-interactions formed by the aforementioned ligands. Other amino acids serving as anchor points in the Src kinase ATP binding pocket include Met341, Ser345, Asp348, and Asp390. The chemical structures of the candidates facilitate hydrophobic interactions with surrounding amino acids, and enable higher ligand-protein affinity and complex stability.

### Bioactivity Prediction by SVM and MLR

The 75 descriptors relevant to the training set were determined and GFA further employed to determine the top ten descriptors that could most accurately describe the training set. The descriptors thus determined were: *C_Count* (the amount of carbon atoms within the ligand), *ES_Sum_sCH_3_* (sum of the electrotopological state (E-state) values for carbons with single bonds), *E_ADJ_equ* (molecule differentiation based on edge adjacency), *CHI_2* (number of pairs of bonds within a molecule), *CHI_3_P* (number of phosphate molecules with three bonds), *Kappa_2* (shape index of order 2 describing topological descriptors), *Kappa_3* (shape index of order 3 describing topological descriptors), *Jurs_FNSA_1* (sum of negative surface areas divided by total molecular solvent-accessible surface area), *Jurs_PNSA_1* (sum of solvent-accessible surface areas of all negatively charged atoms), *Shadow_nu* (characterizes molecule shape using ratio of largest to smallest dimension).

**Figure 7:**
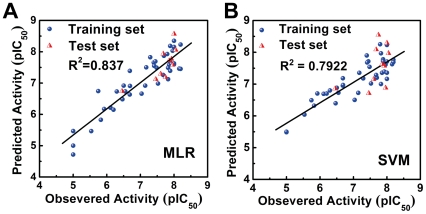
Correlation between observed and predicted activities of 53 Src inhibitors using different prediction models. (A) MLR and (B) SVM. Correlation coefficients (R^2^) calculated from the training set were 0.837 and 0.7922 for MLR and SVM models respectively.

Using these descriptors, the MLR model generated was:
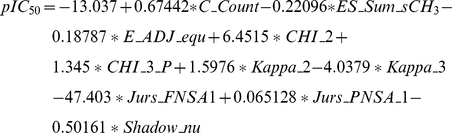



As shown in Figure 7, the square correlation coefficients for the MLR and SVM models were 0.837 and 0.7922, respectively, indicating reliable models. Predicted bioactivities (pIC_50_) of the TCM candidates by the generated MLR and SVM models are listed in Table 2. Both models predicted higher pIC_50_ values for Isopraeroside IV and 9HGF than aurantiamide. The predicted bioactivities for Saracatinib was 4.9410 using MLR and 7.3036 using SVM. These predicted results, when compared to the *in vitro* inhibition concentration of Saracatinib (pIC_50_ = 8.57) [47], indicate that values predicted by the SVM model may be closer to actual inhibition concentrations observed *in vitro*. The higher accuracy of the SVM model is expected as its predictions are generated on a non-linear model.

### Bioactivity Prediction by 3D-QSAR

Table 4 summarizes the PLS results of CoMFA and CoMSIA models. Steric fields are the dominant factor in CoMFA. Under an optimal number of components (ONC) of 6, cross-validated correlation coefficient (q^2^) and non-cross-validated correlation coefficient (r^2^) of 0.430 and 0.920 indicate a reliable model. Multiple CoMSIA models were generated using different factor combinations (Table 4). Models SHD and SEHD were the best overall models with high q^2^ (q^2^>0.4), high r^2^, low SEE, and large F values. However, addition of an electrostatic variable in model SEHD did not significantly increase model correlation or strength (F-value), thus model SHD was selected as the optimum CoMSIA model. Reliability of the selected CoMFA and CoMSIA models are validated by the q^2^ and r^2^ values (Table 4).

**Table 4:** CoMFA and CoMSIA models constructed from 53 known Src kinase inhibitors^1^.

	Cross validation		Non-cross validation			Fraction				
	ONC	q^2^cv	r^2^	SEE	F	S	E	H	D	A
CoMFA	6	0.430	0.920	0.291	68.862	1	0	-	-	-
CoMSIA										
S	6	0.567	0.879	0.357	43.751	1	-	-	-	-
H	6	0.449	0.922	0.287	70.832	-	-	1	-	-
SA	5	0.370	0.821	0.428	34.018	0.587	-	-	-	0.413
SE	5	0.468	0.845	0.399	40.426	1	0	-	-	-
SH	6	0.486	0.935	0.262	86.523	0.367	-	0.633	-	-
HD	6	0.499	0.929	0.273	78.719	-	-	0.695	0.305	-
SEH	5	0.527	0.914	0.298	78.403	0.368	0	0.632	-	-
SHD*	6	0.519	0.944	0.244	100.517	0.260	-	0.468	0.272	-
SHA	6	0.439	0.936	0.260	87.726	0.293	-	0.492	-	0.215
EHD	6	0.531	0.929	0.273	78.719	-	0	0.695	-	0.305
HDA	5	0.446	0.928	0.275	77.512	-	-	0.550	0.253	0.197
SEHD	6	0.539	0.944	0.244	100.517	0.261	0	0.468	0.272	-
SEHA	5	0.506	0.920	0.286	85.421	0.287	0	0.485	-	0.228
SHDA	6	0.467	0.946	0.238	105.771	0.222	-	0.400	0.219	0.158
EHDA	6	0.464	0.928	0.275	77.512	-	0	0.550	0.253	0.197
SEHDA	6	0.452	0.946	0.238	105.771	0.222	0	0.400	0.219	0.158

1 : Structures and inhibitory activities adapted from [54].

ONC: Optimal number of components.

S: Steric.

SEE: Standard error of estimate.

E: Electrostatic.

F: F-test value.

H: Hydrophobic.

PLS: partial least squares.

D: Donor.

* : Optimum prediction model.

A: Acceptor.

**Figure 8:**
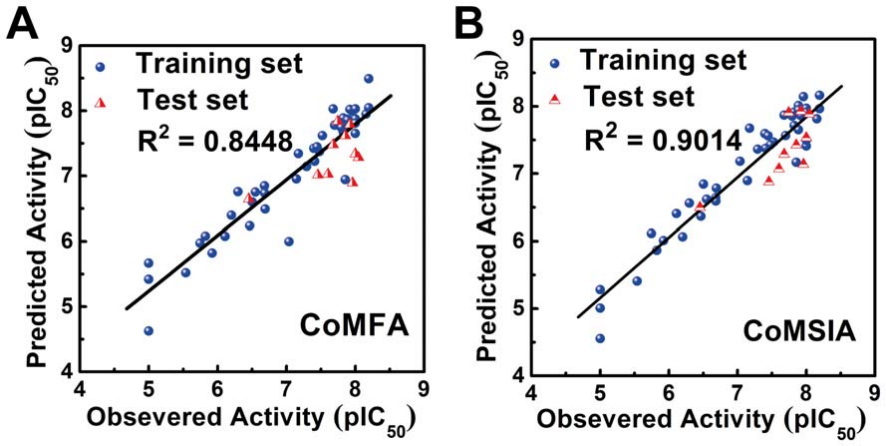
Correlation between observed and predicted activities of 53 Src inhibitors using different 3D-QSAR models. (A) CoMFA and (B) CoMSIA. Correlation coefficients (R^2^) calculated from the training set were 0.8448 and 0.9014 for CoMFA and CoMSIA models respectively.

The observed, predicted and calculated residual pIC_50_ values of the training and test set compounds by CoMFA and CoMSIA are summarized in Table S1. Differences between observed and predicted pIC_50_ ranged between −0.667 and 1.066. Residuals using the CoMSIA model ranged from −0.5 to 0.821, indicating high accuracy of the predictions from our generated model with actual biologically verified values. All ratio values calculated through CoMSIA were within 1±0.1 indicating high accuracy. The correlation coefficient between the observed and predicted pIC_50_ using the CoMFA model was 0.8448 (Figure 8A). With the CoMSIA model, the correlation coefficient was 0.9014 (Figure 8B). Both models are highly reliable.

**Figure 9:**
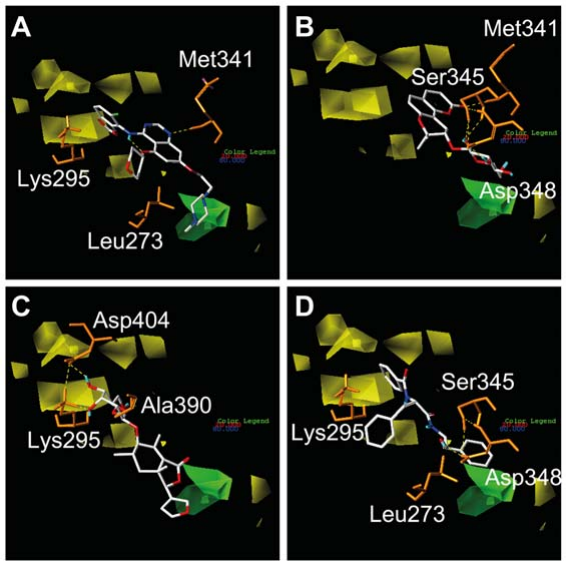
Spatial contour of test ligands to generated CoMFA maps. Map areas favoring and disfavoring steric fields are represented respectively in green and yellow. Saracatinib (A), Isopraeroside IV (B), and 9HGF (C) contoured well to the bioactivity map. A benzene ring of aurantiamide was located within the steric disfavoring region (D), suggesting lower bioactivity than it other tested ligands.

**Figure 10:**
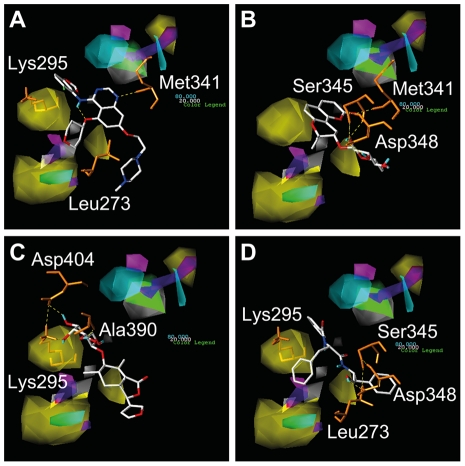
Spatial contour of test ligands to generated CoMSIA maps. Color blocks represent favor/disfavor steric field (green/yellow), favor/disfavor hydrophobic interaction (cyan/white), favor/disfavor hydrogen donor (purple/magenta). Saracatinib (A) forms H-bonds with Met341 which is located near the region favoring hydrogen bond donors and hydrophobic interactions. The compact structures of Isopraeroside IV (B) and 9HGF (C) allowed interactions with Src kinase without defying regions disfavoring interactions. The benzene moieties of aurantiamide (D) were in proximity to the steric disfavoring region, suggesting lower bioactivity.

Superimposing CoMFA and CoMSIA contour maps on Saracatinib and the TCM candidates provide insights into the predicted bioactivity of the compounds. Saracatinib (Figure 9A), Isopraeroside IV (Figure 9B), and 9HFG (Figure 9C) contoured well to the CoMFA model. No bulk structures were located in the steric disfavoring (yellow) region. In addition, bulky ring moieties in Saracatinib and 9HFG were located in the steric favoring (green) region. By comparison, a benzene ring of aurantiamide was located within the steric disfavoring region, suggesting a lower biological activity due to its deviation from the CoMFA contour map (Figure 9D). These results agree with the MLR and SVM results in which aurantiamide had the lowest predicted bioactivity among the tested compounds. With regard to CoMSIA maps, Saracatinib forms H-bonds with Met341 which is located near the region favoring hydrogen bond donors (purple) and hydrophobic interactions (cyan) (Figure 10A). Isopraeroside IV was a smaller compound and its interactions with Src did not fall within the disfavoring regions of the CoMSIA map (Figure 10B). A similar contour was observed in 9HFG (Figure 10C). In aurantiamide, the benzene moieties were in proximity to the steric disfavoring regions, suggesting a lower bioactivity of the ligand (Figure 10D). Results of the ligand-based studies are consistent, and suggest high bioactivity for Saracatinib, moderate to high bioactivity for Isopraeroside and 9HFG, and moderate to low bioactivity for aurantiamide.

### Molecular Dynamics Simulation

**Figure 11:**
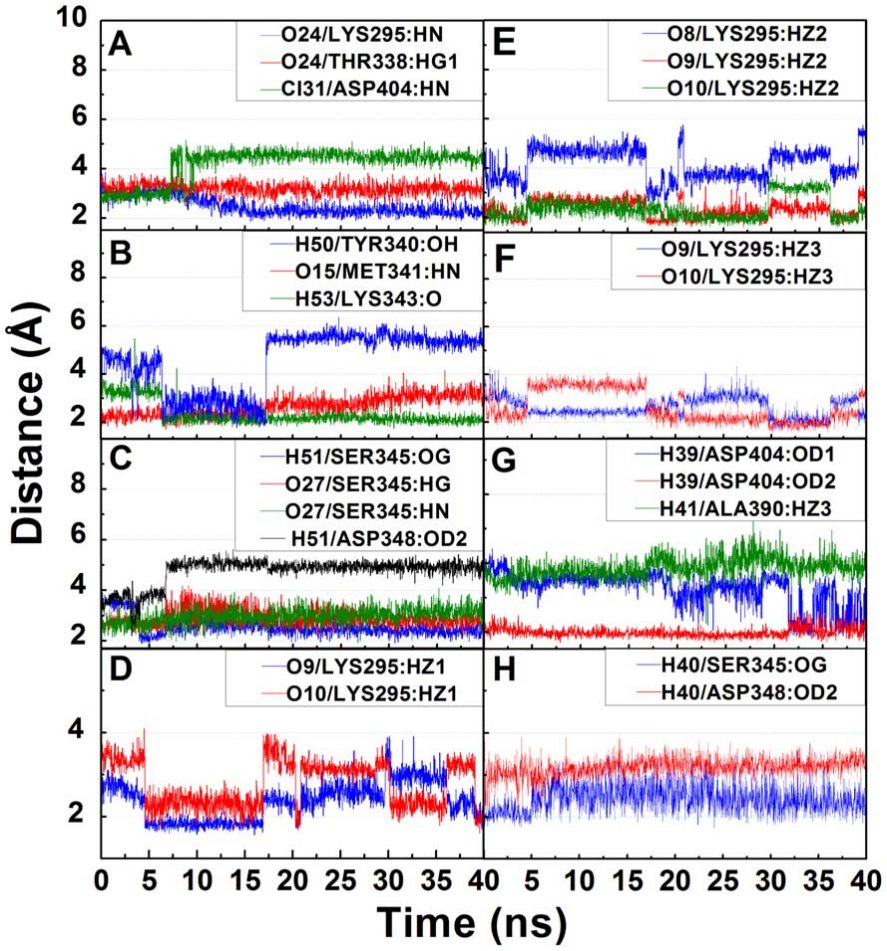
Hydrogen bond distance profiles between Src kinase and TCM candidates during 20 ns MD simulation. Distances (Å) shown are profiles of amino acids implicated as binding residues during docking with (A) Saracatinib, (B,C) Isopraeroside IV, (D–G) 9HFG, and (H) aurantiamide.

**Figure 12:**
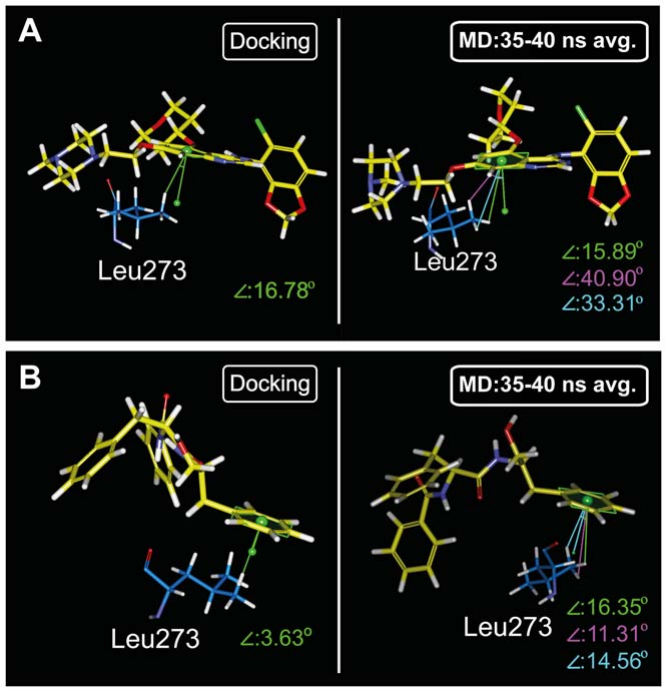
Molecular changes contributing to the disappearance of pi interactions in Saracatinib and aurantiamide during MD. Loss of pi-interaction with Leu273 in both ligands was due to torsion of the benzene ring within the ligand structures.

More realistic interactions of the candidates with Src kinase in a biological system were simulated through MD. Interaction differences at key residues for each tested compound during docking and MD are summarized in Table 5. H-bond formation and occupancies during and the H-bond distance profiles are presented in Table 6 and Figure 11, respectively. The primary binding residue for Saracatinib was Lys295 where stable π-interactions and a high occupancy H-bond were formed (Figure 11A). The H-bond observed at Met341 during docking was substituted by an H-bond with Thr338 throughout MD. The π interaction with Leu273 during docking was not observed during MD. As illustrated in Figure 12A, the head-on direction of the H atom and the near perpendicular spatial arrangements facilitate the formation of π-sigma interaction between Leu273 and Saracatinib. During MD, angle increase due to ligand and Leu273 fluctuations disrupted the formation of π-sigma interactions (Figure 12A).

**Table 5:** Summary of interaction type, location, and frequency of test ligands following docking and MD simulation.

Ligand	Interaction Location/Type/Frequency*									
	Leu273	Lys295	Thr338	Tyr340	Met341	Lys343	Ser345	Asp348	Ala390	Asp404
Saracatinib-docking	π	π			H					
Saracatinib-MD		πH	H		H					
Isopraeroside IV-docking					H		HH	HH		
Isopraeroside IV-MD				H	H		HHH			
9HFG		HHH							H	H
9HFG-MD		HHHHHH								HH
Aurantiamide	π	π					H	H		
Aurantiamide-MD		π					H	H		

* : each letter denotes one interaction.

π: pi-interaction.

H: H-bond.

**Table 6:** H-bond distance and occupancies^1^ of different ligands during MD.

Ligand	H-bond	Ligand Atom	Amino acid	Distance (Å)			H-bond occupancy
				Max.	Average	Min.	
Saracatinib	1	O24	LYS295:HN	3.865	2.492	1.863	59.8%
	2	O24	THR338:HG1	3.875	3.198	2.296	0.1%
	3	Cl31	ASP404:HN	5.164	4.193	2.480	0.15%
Isopraeroside IV	1	H50	TYR340:OH	6.354	4.565	1.773	7.75%
	2	O15	MET341:HN	4.145	2.655	1.714	39.45%
	3	H53	LYS343:O	5.479	2.331	1.746	49.25%
	4	H51	SER345:OG	3.933	2.601	1.896	70.55%
	5	O27	SER345:HG	4.195	2.894	2.165	7.85%
	6	O27	SER345:HN	4.094	2.976	1.929	6.7%
	7	H51	ASP348:OD2	5.625	4.741	2.193	15.3%
9HFG	1	O9	LYS295:HZ1	3.92	2.358	1.565	60.2%
	2	O10	LYS295:HZ1	4.099	2.773	1.61	40.6%
	3	O8	LYS295:HZ2	5.772	4.166	2.451	0.05%
	4	O9	LYS295:HZ2	3.31	2.327	1.601	63.95%
	5	O10	LYS295:HZ2	3.741	2.350	1.592	71.15%
	6	O9	LYS295:HZ3	4.354	2.627	1.633	42.85%
	7	O10	LYS295:HZ3	4.102	2.637	1.562	57.55%
	8	H41	ALA390:O	5.284	3.869	2.186	0.05%
	9	H39	ASP404:OD1	4.427	3.246	1.784	14.35%
	10	H39	ASP404:OD2	4.427	3.246	1.784	99.4%
Aurantiamide	1	H40	SER345:OG	3.764	2.431	1.743	61.5%
	2	H44	ASP348:OD1	3.896	3.182	2.151	0.9%

1 : Occupancy calculated based on the default cut-off distance of 2.5 Å.

**Figure 13:**
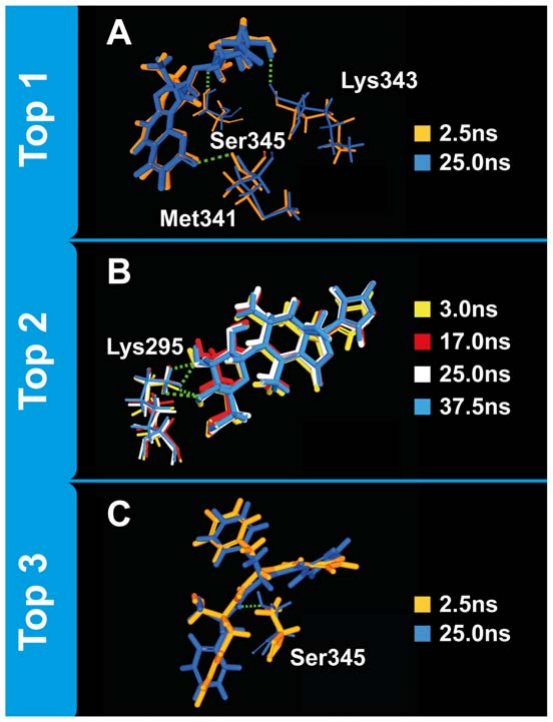
Molecular changes contributing to protein-ligand complex stability during MD. (A) Lys343 and Ser345 amine group rotations bring Isopraeroside IV into proximity for H-bond formation. (B) The rhythmic fluctuations observed in 9HFG can be attributed to the rotations of the NH_3_ group on Lys295. (C) Rotation of the benzene moieties led to increased distance fluctuations in Ser345 during MD.

Isopraeroside IV formed stable H-bonds with Met341, Lys343, and Ser345 (Table 6). H-bonds formed with Tyr340 (Figure 11B) and Asp348 (Figure 11C) were not stable and could not contribute to stability of Isopraeroside when in complex with Src kinase. Sharp decreases in H-bond distances observed for Lys343 and Ser345 (Figure 11B–C) are likely due to rotations of the residues which bring the ligand into closer proximity for H-bond formation (Figure 13A).

The highest number of H-bonds was formed by 9HFG with Lys295 and Asp404 (Table 5–6) Rhythmic fluctuations of H-bond distances with the key binding residue Lys295 (Figure 11D–F) are likely due to rotations of the amine group on Lys295 (Figure 13B). 9HFG also formed two H-bonds with Asp404, but the H-bond with Ala390 during docking was lost (Figure 11G).

Aurantiamide was primarily bound to the ATP binding pocket through the π interaction at Lys295 and H-bonds with Ser345 and Asp348 (Table 5). Increased distance fluctuations in Ser345 (Figure 11H) could be due to the rotation of benzene moieties (Figure 13C). Similar to Saracatinib, the loss of the π interaction at Leu273 involved torsion of the benzene moiety (Figure 12B). Benzene ring torsion during MD reduced angles between the H atom and the benzene normal plane to approximately 90 degrees, making the formation of π-interactions unlikely (Figure 12B). In general, MD results were in agreement with docking (Table 5).

**Figure 14:**
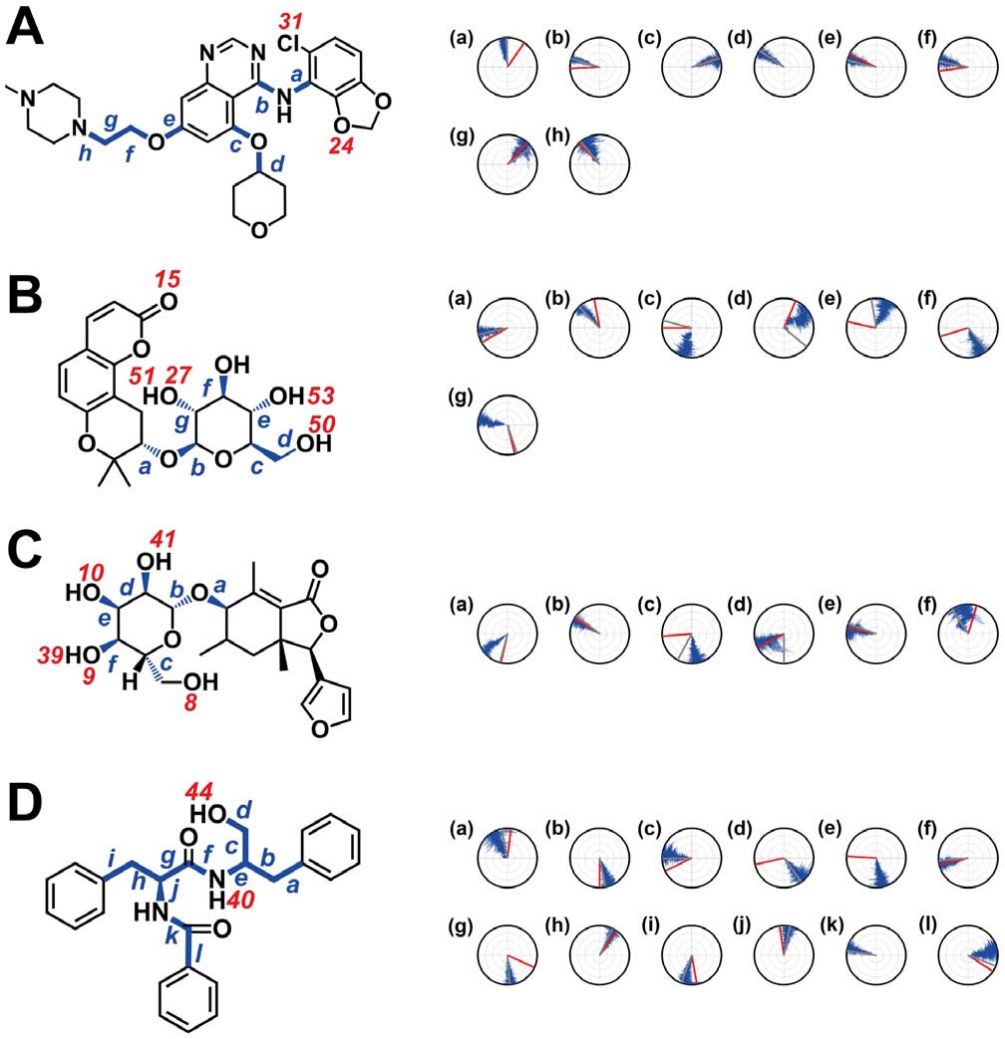
Torsion angles of test candidates during MD simulation. Torsion angle measured is designated by a lower case alphabet which corresponds to the radar chart with the same alphabet. Red numbers indicate the locations where H-bonds are formed. (numbering corresponds to those in Table 5). Blue lines indicate the torsion angles recorded; the red and gray lines indicate the angle during docking and at time 0 of MD, respectively.

Torsion angles also provide molecular insights into ligand stability (Figure 14). Torsion angle fluctuations critical to the stability of Saracatinib were not observed (Figure 14A). Torsion angles recorded for Isopraeroside IV were relatively stable with the exception of **c** and **d** (Figure 14B). Notable torsion angle changes at **c** and **d**, may account for the decrease in H-bond distances observed for Tyr340 (6.34 ns) (Figure 11B). Subsequent increase in bond distance and resulting loss of the Tyr340 H-bond could be due to sharp angle changes of **d** from 17.44 ns to the end of MD simulation. Torsion angles for 9HFG provide supporting evidence for the cause of H-bond fluctuations in Figure 11D–G. Since the stable torsion angles at **c** and **e** (Figure 14C) imply a relatively stable ligand, fluctuations in bond distance (Figure 11D–F) should be due to changes in Lys295. Further evidence is given by torsion angle changes at **f**. As **f** is associated with the binding of O9 with Lys295 and H39 with Asp404, should ligand stability be the cause of fluctuation, bond distances at both locations should be equally affected. However, H39 forms stable H-bond with Asp404, but O9 does not form stable bonding with Lys295, validating that the primary reason for the lack of stable binding with Lys295 is due to the instability of the amino acid itself. Among the torsion angles recorded for aurantiamide (Figure 14D), only **a**, **d**, and **l** are relatively unstable with angle fluctuations greater than 50 degrees. Stable H-bonds are formed as a result of the stability of the ligand.

**Figure 15:**
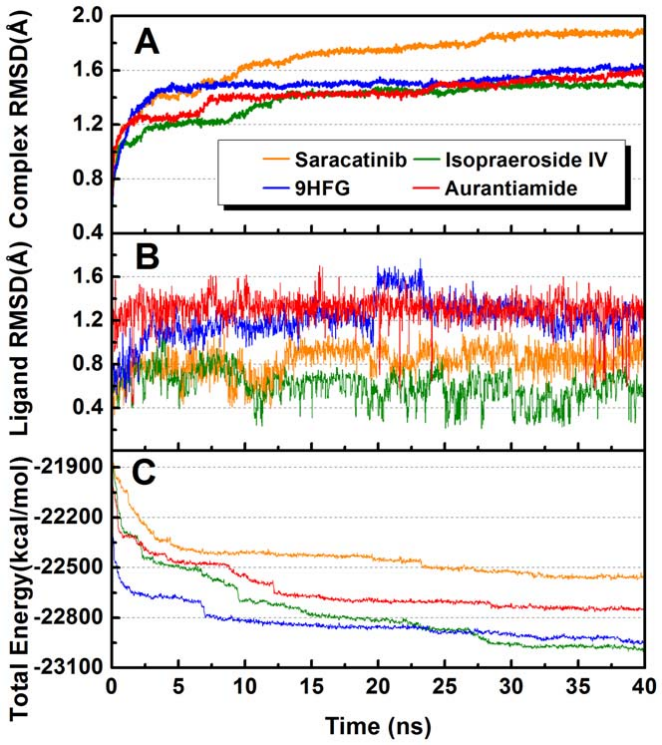
Measured trajectories during MD. (A) Complex, (B) ligand, and (C) total energy during MD. Saracatinib had the highest complex RMSD and total energy. The lower complex RMSDs and total energy of Isopraeroside IV and 9HFG may be attributed to their compact structure and multiple H-bond formations. The higher RMSD and total energy observed in auranruamide is possibly the result of the rotating benzene moieties.

Figure 15 summarizes the RMSD and total energy trajectories of the ligands during MD. All compounds, with the exception of 9HFG from 20–24 ns, had relatively stable ligand RMSDs. Whole molecule RMSDs and total energies reached equilibration after 16 ns. Among the four test compounds, Saracatinib had the highest complex RMSD and total energy. The TCM candidates exhibited similar complex RMSDs, but aurantuamide had higher total energy than Isopraeroside IV and 9HFG after stabilization. The higher energy and complex RMSD of Saracatinib may be associated with its high internal ligand energy (Table 1). Once bound, the internal energy is transferred to the complex, causing more fluctuations and higher energy levels. Isopraeroside IV and 9HFG are smaller structures compared to Saracatinib. The lower energy profiles and complex RMSD may be attributed to the compact structures and formation of multiple H-bonds. Flexible moieties in Isopraeroside IV are anchored to the ATP binding pocket through H-bonds, limiting the fluctuations of the complex and increasing stability. By contrast, H-bonds formed by 9HFG stabilizes the phenyl moiety, but the remaining structure is unbound and can freely rotate. This could be the reason for high ligand RMSDs observed for 9HFG (Figure 15B). Aurantuamide is a larger compound that shares similarities with Saracatinib. As previously discussed, aurantuamide interacts with Src kinase ATP binding pocket through a π interaction with Lys295 and an H-bond at Ser345, leaving two benzene moieties free for rotation. The larger ligand RMSD and higher total energy is likely due to these rotations. In summary, all four test compounds formed stable complexes at the ATP binding pocket for the duration of MD. TCM candidates exhibited higher stability than Saracatinib, indicating advantages as substitutes for the commercial drug.

### Assessment of TCM candidate mode of action

**Figure 16:**
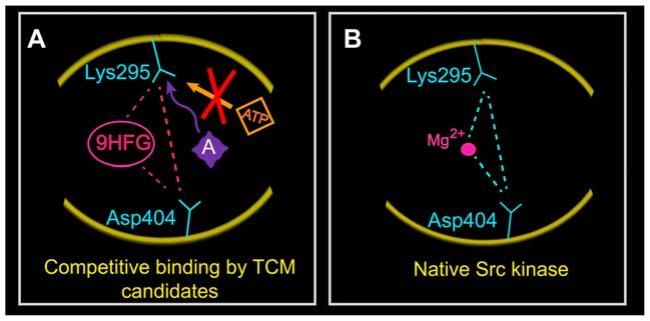
Mechanisms underlying Src kinase inhibition and activation. (A) Based on the molecular interactions observed through MD, TCM candidates 9HFG and aurantiamide (A; violet diamond) may inhibit Src kinase activation through different pathways. Aurantiamide binds directly to Lys295, competing with ATP for the binding site. 9HGF also binds to Lys295, but has additional binding with Asp404, which acts similarly to Mg^2+^ found in native Src kinase. (B) The bridging function of Mg^2+^ between Lys295 and Asp404 in native Src kinase.

Despite promising drug-like characteristics, one must consider binding mechanisms of the ligand compounds to objectively speculate their effect on Src kinase. As described previously, most Src inhibitors, including Saracatinib, compete with ATP for binding to Lys295 (Type I inhibitors). Src kinase is activated when interactions between SH2 and SH3 are broken, and when Tyr416 becomes phosphorylated [18] (Figure 1). Among our TCM candidates, 9HFG and aurantiamide show Src Type I inhibitor-like docking characteristics. Aurantiamide interacts primarily with Lys295, and thus its mode of inhibition would also be through competitive binding against ATP for Lys295 (Figure 16A). However, its lower total energy (Figure 15) implies a more stable protein-ligand complex than Saracatinib. Intriguingly, 9HFG is a potential candidate that mimics the natural autoinhibitory mechanisms in Src kinases. Not only does 9HFG bind strongly to Lys295, but it simultaneously binds to Asp404 as well (Figure 16A). This is an important feature as it mimics the bridging function of Mg^2+^ between these two amino acids in native Src kinases and stabilizes the structural integrity of the catalytic cleft (Figure 16B) [18]. The dual function of 9HFG as a binding site competitor and stabilizer of the catalytic cleft suggests good potential for Src kinase inhibition. On the contrary, Isopraeroside IV may not affect Src kinase activity despite its good binding affinity and predicted bioactivities. Isopraeroside IV binds to regions located deeper within the catalytic cleft than Lys295 (Table 3, Table 5) and does not interact directly with Lys295. Though Isopraeroside IV may limit the ability of ATP to bind to Lys295 through steric hindrance, there is no evidence from our structure-based analysis to support such a claim.

### Conclusion

The potential of Isopraeroside IV, 9HFG, and aurantiamide as potential leads for Src-kinase was assessed through structure- and ligand-based *in silico* approaches. Docking and MD suggest that Isopraeroside IV, 9HFG, and aurantiamide have higher and more stable binding affinities with Src kinase than Saracatinib. Assessment of biological activity through ligand structures further implied that the TCM candidates were biologically active compounds. Considering the interaction between the TCM candidates and the Src ATP binding region, 9HFG, and aurantiamide may exert inhibition against Src kinase by competitive binding against ATP. Our results may be applicable to drug development in different ways. TCM compounds 9HFG and aurantiamide may be directly used as candidate lead compounds in biological studies based on their high stability and predicted bioactivities. In addition, structural insights such as key amino acids and important moieties which form stable interactions can be utilized for *de novo* synthesis of more stable compounds that interact with Src kinase.

## Materials and Methods

### Docking

The protein structure of human Src kinase (PDB: 2H8H) was obtained from Protein Data Bank [47]. The binding site used in this study was based on the space occupied by Saracatinib within 2H8H. The binding site was located within the cleft separating the carboxyl-terminal lobe (residues 345–523) and the amino-terminal lobe (residues 270–340), and surrounded by residues Lys295, Trp340, Met341, Lys343, Gly344, Ser345, Leu373, Leu393, and Asp404. Over 20,000 ligands from TCM Database@Taiwan were used for docking in the Src kinase binding site. All ligands were pre-treated with CHARMm [48] to attach missing H-atoms. Saracatinib was used as the control. TCM ligands were docked into the binding site using the LigandFit program [49] within Discovery Studio 2.5 and DockScore selected as the primary scoring function for assessing binding affinities. TCM ligands were further screened with Lipinski's Rule of Five [50], [51] and absorption, distribution, metabolism, excretion and toxicity (ADMET) [52] in DS 2.5 to rule out potentially toxic derivatives. Attraction forces (H-bond and hydrophobic interactions) between the ligand and protein were analyzed using LigPlot v.2.2.25 [53]. The 3D protein-ligand complexes generated during docking were flattened to 2D diagrams through the ligplot algorithm.

### Bioactivity Prediction by Support Vector Machine (SVM) and Multiple Linear Regression (MLR)

DS 2.5 was used to calculate individual molecular property descriptors of 53 Src inhibitors with known pIC_50_ values [54]. Representative descriptors for the inhibitors were determined from a pool of 552 descriptors through genetic function approximation (GFA) [55], [56]. A QSAR models were calculated by GFA and ranked by square correlation coefficient (R^2^) corresponding to the fitness of each model. Descriptors from the model with the highest R^2^ were used to construct MLR and SVM models for predicting the bioactivity of TCM candidates. The linear MLR model [57] was constructed with the representative descriptors using MATLAB [58].

In addition to the linear MLR model, a non-linear QSAR model utilizing support vector machine (SVM) [59], [60] for the regression of continuous bioactivity data (pIC_50_), also termed support vector regression (SVR), was also constructed. SVR uses the Kernel function to map the input data set into a high-dimensional feature space in order to find the hyperplane that best predicts data distribution. The SVR formulation is
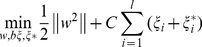
(1)

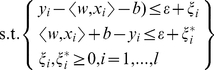
(2)The parameters (*x*_1_, *y*_1_), …, (*x*_l_, *y*_l_) represent data within a given training set and can also be expressed as *f*(x) = *<w,x*>+*b*, 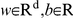. The parameter *w* is calculated by performing ε-insensitive loss function. The performance of SVR is dependent on parameters *C*,ε, the kernel function and kernel parameters. To further optimize this to nonlinear functions, Lagrange multipliers and kernel *k*(*x*,*x*′) = 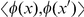 are used. The nonlinear functions and predictions are determined by:
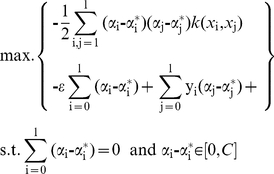
(3)

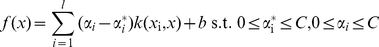
(4)The SVM model was constructed by applying the LibSVM [61] within the gridregression.py program to determine key parameters C cost, epsilon and gamma.

### Bioactivity Prediction by 3D Quantitative Structure-Activity Relationship (QSAR) Analysis

The 53 Src kinase inhibitors [54] used in this study were randomly divided into a training set of 43 compounds and a test set of 10 compounds. Alignment of the training set molecules was performed using atom-fit module of SYBYL-X 1.1 [62] Comparative force field analysis (CoMFA) and comparative similarity indices analysis (CoMSIA) models were constructed for 3D-QSAR. The steric and electrostatic field descriptors in CoMFA were calculated using Lennard-Jones potential and Coulombic potential, respectively. For CoMSIA models, the steric, electrostatic, hydrophobic and hydrogen bond donor and acceptors were calculated through Gaussian functions. Partial least squares (PLS) regression was utilized to analyze the 3D-QSAR models with descriptor used as an independent variable. Structural contour to the generated CoMFA and CoMSIA models were used to predict bioactivity of TCM candidates.

### Molecular Dynamics (MD) Simulation

Src kinase complexes of the TCM candidates were subjected to molecular dynamics (MD) simulation under the force field of CHARMm [48] using DS 2.5. The energy of each complex was minimized using 500 steps of Steepest Descent and 500 steps of Conjugate Gradient method. The system was heated without constraint from 50 K to 310 K in 50 ps, and equilibrated for 200 ps. The final production was conducted for 40 ns in NVT ensemble with snapshots saved at 2.5 ps intervals. The time step was set to 2 fs. Hydrogen bond frequencies, energy trajectories, and hydrogen bond distances were calculated via DS 2.5 Analyze Trajectory module to analyze molecular interactions within the protein-ligand system.

## Supporting Information

Table S1 Predicted and observed activity (pIC_50_) of 53 known Src kinase inhibitors^1^ using generated CoMFA and CoMSIA models.
(DOC)

## References

[1] Wheeler DL, Iida M, Dunn EF (2009). The role of Src in solid tumors.. Oncologist.

[2] Oneyama C, Morii E, Okuzaki D, Takahashi Y, Ikeda J (2011). MicroRNA-mediated upregulation of integrin-linked kinase promotes Src-induced tumor progression.. Oncogene.

[3] Gianni D, Taulet N, DerMardirossian C, Bokoch GM (2010). c-Src-mediated phosphorylation of NoxA1 and Tks4 induces the reactive oxygen species (ROS)-dependent formation of functional invadopodia in human colon cancer cells.. Mol Biol Cell.

[4] Miyake T, Parsons SJ (2011). Functional interactions between Choline kinase alpha, epidermal growth factor receptor and c-Src in breast cancer cell proliferation.. Oncogene.

[5] Irwin ME, Bohin N, Boerner JL (2011). Src family kinases mediate epidermal growth factor receptor signaling from lipid rafts in breast cancer cells.. Cancer Biol Ther.

[6] Thomas S, Overdevest JB, Nitz MD, Williams PD, Owens CR (2011). Src and caveolin-1 reciprocally regulate metastasis via a common downstream signaling pathway in bladder cancer.. Cancer Res.

[7] Kim HS, Han HD, Armaiz-Pena GN, Stone RL, Nam EJ (2011). Functional roles of Src and Fgr in ovarian carcinoma.. Clin Cancer Res.

[8] Ferrandi M, Molinari I, Torielli L, Padoani G, Salardi S (2010). Adducin- and ouabain-related gene variants predict the antihypertensive activity of rostafuroxin, part 1: experimental studies.. Sci Transl Med.

[9] Ferrari P (2010). Rostafuroxin: an ouabain-inhibitor counteracting specific forms of hypertension.. Biochim Biophys Acta.

[10] Manunta P, Ferrandi M, Bianchi G, Hamlyn JM (2009). Endogenous ouabain in cardiovascular function and disease.. J Hypertens.

[11] Bianchi G (2005). Genetic variations of tubular sodium reabsorption leading to “primary” hypertension: from gene polymorphism to clinical symptoms.. Am J Physiol Regul Integr Comp Physiol.

[12] Ferrandi M, Molinari I, Barassi P, Minotti E, Bianchi G (2004). Organ hypertrophic signaling within caveolae membrane subdomains triggered by ouabain and antagonized by PST 2238.. J Biol Chem.

[13] Scheiner-Bobis G, Schoner W (2001). A fresh facet for ouabain action.. Nat Med.

[14] Liu J, Tian J, Haas M, Shapiro JI, Askari A (2000). Ouabain interaction with cardiac Na+/K+-ATPase initiates signal cascades independent of changes in intracellular Na+ and Ca2+ concentrations.. J Biol Chem.

[15] Ferrandi M, Salardi S, Tripodi G, Barassi P, Rivera R (1999). Evidence for an interaction between adducin and Na(+)-K(+)-ATPase: relation to genetic hypertension.. Am J Physiol-Heart C.

[16] Aleshin A, Finn RS (2010). SRC: A Century of Science Brought to the Clinic.. Neoplasia.

[17] Roskoski R (2004). Src protein-tyrosine kinase structure and regulation.. Biochem Biophys Res Commun.

[18] Xu W, Doshi A, Lei M, Eck MJ, Harrison SC (1999). Crystal structures of c-Src reveal features of its autoinhibitory mechanism.. Mol Cell.

[19] Zhang J, Yang PL, Gray NS (2009). Targeting cancer with small molecule kinase inhibitors.. Nat Rev Cancer.

[20] Liu Y, Gray NS (2006). Rational design of inhibitors that bind to inactive kinase conformations.. Nat Chem Biol.

[21] Cortes JE, Kantarjian HM, Brummendorf TH, Kim DW, Turkina AG (2011). Safety and efficacy of bosutinib (SKI-606) in chronic phase Philadelphia chromosome-positive chronic myeloid leukemia patients with resistance or intolerance to imatinib.. Blood.

[22] Kluger HM, Dudek AZ, McCann C, Ritacco J, Southard N (2011). A Phase 2 Trial of Dasatinib in Advanced Melanoma.. Cancer-Am Cancer Soc.

[23] Mayer EL, Baurain JF, Sparano J, Strauss L, Campone M (2011). A Phase 2 Trial of Dasatinib in Patients with Advanced HER2-Positive and/or Hormone Receptor-Positive Breast Cancer.. Clin Cancer Res.

[24] Herold CI, Chadaram V, Peterson BL, Marcom PK, Hopkins J (2011). Phase II Trial of Dasatinib in Patients with Metastatic Breast Cancer Using Real-Time Pharmacodynamic Tissue Biomarkers of Src Inhibition to Escalate Dosing.. Clin Cancer Res.

[25] Sharma MR, Wroblewski K, Polite BN, Knost JA, Wallace JA (2011). Dasatinib in previously treated metastatic colorectal cancer: a phase II trial of the University of Chicago Phase II Consortium.. Invest New Drugs.

[26] Johnson ML, Riely GJ, Rizvi NA, Azzoli CG, Kris MG (2011). Phase II trial of dasatinib for patients with acquired resistance to treatment with the epidermal growth factor receptor tyrosine kinase inhibitors erlotinib or gefitinib.. J Thorac Oncol.

[27] Brooks HD, Glisson BS, Bekele BN, Ginsberg LE, El-Naggar A (2011). Phase 2 study of dasatinib in the treatment of head and neck squamous cell carcinoma.. Cancer-Am Cancer Soc.

[28] Finn RS, Bengala C, Ibrahim N, Roche H, Sparano J (2011). Dasatinib as a Single Agent in Triple-Negative Breast Cancer: Results of an Open-Label Phase 2 Study.. Clin Cancer Res.

[29] Johnson FM, Bekele BN, Feng L, Wistuba I, Tang XM (2010). Phase II Study of Dasatinib in Patients With Advanced Non-Small-Cell Lung Cancer.. J Clin Oncol.

[30] Fury MG, Baxi S, Shen RL, Kelly KW, Lipson BL (2011). Phase II Study of Saracatinib (AZD0530) for Patients with Recurrent or Metastatic Head and Neck Squamous Cell Carcinoma (HNSCC).. Anticancer Res.

[31] Mackay HJ, Au HJ, McWhirter E, Alcindor T, Jarvi A (2011). A phase II trial of the Src kinase inhibitor saracatinib (AZD0530) in patients with metastatic or locally advanced gastric or gastro esophageal junction (GEJ) adenocarcinoma: a trial of the PMH phase II consortium.. Invest New Drugs.

[32] Gucalp A, Sparano JA, Caravelli J, Santamauro J, Patil S (2011). Phase II trial of saracatinib (AZD0530), an oral SRC-inhibitor for the treatment of patients with hormone receptor-negative metastatic breast cancer.. Clin Breast Cancer.

[33] Chen CYC (2011). TCM Database@Taiwan: the world's largest traditional Chinese medicine database for drug screening in silico.. PLoS One.

[34] Tsai TY, Chang KW, Chen CYC (2011). iScreen: world's first cloud-computing web server for virtual screening and de novo drug design based on TCM database@Taiwan.. J Comput Aided Mol Des.

[35] Chang KW, Tsai TY, Chen KC, Yang SC, Huang HJ (2011). iSMART: an integrated cloud computing web server for traditional Chinese medicine for online virtual screening, de novo evolution and drug design.. J Biomol Struct Dyn.

[36] Yang SC, Chang SS, Chen CYC (2011). Identifying HER2 Inhibitors from Natural Products Database.. PLoS One.

[37] Yang SC, Chang SS, Chen HY, Chen CYC (2011). Identification of potent EGFR inhibitors from TCM Database@Taiwan.. PLoS Comput Biol.

[38] Sun MF, Chang TT, Chang KW, Huang HJ, Chen HY (2011). Blocking the DNA repair system by traditional Chinese medicine?. J Biomol Struct Dyn.

[39] Chang SS, Huang HJ, Chen CYC (2011). Two Birds with One Stone? Possible Dual-Targeting H1N1 Inhibitors from Traditional Chinese Medicine.. PLoS Comput Biol.

[40] Chen KC, Chen CYC (2011). Stroke prevention by traditional Chinese medicine? A genetic algorithm, support vector machine and molecular dynamics approach.. Soft Matter.

[41] Chang TT, Sun MF, Chen HY, Tsai FJ, Fisher M (2011). Screening from the world's largest TCM database against H1N1 virus.. J Biomol Struct Dyn.

[42] Chang PC, Wang JD, Lee MM, Chang SS, Tsai TY (2011). Lose weight with traditional Chinese medicine? Potential suppression of fat mass and obesity-associated protein.. J Biomol Struct Dyn.

[43] Chen CY, Chen CYC (2010). Insights into designing the dual-targeted HER2/HSP90 inhibitors.. J Mol Graph Model.

[44] Chen CY, Chang YH, Bau DT, Huang HJ, Tsai FJ (2009). Discovery of potent inhibitors for phosphodiesterase 5 by virtual screening and pharmacophore analysis.. Acta Pharmacol Sin.

[45] Choi SY, Ahn EM, Song MC, Kim DW, Kang JH (2005). In vitro GABA-transaminase inhibitory compounds from the root of Angelica dahurica.. Phytother Res.

[46] Yoon JS, Sung SH, Kim YC (2008). Neuroprotective limonoids of root bark of Dictamnus dasycarpus.. J Nat Prod.

[47] Hennequin LF, Allen J, Breed J, Curwen J, Fennell M (2006). N-(5-chloro-1,3-benzodioxol-4-yl)-7-[2-(4-methylpiperazin-1-yl)ethoxy]-5- (tetrahydro-2H-pyran-4-yloxy)quinazolin-4-amine, a novel, highly selective, orally available, dual-specific c-Src/Abl kinase inhibitor.. J Med Chem.

[48] Brooks BR, Brooks CL, Mackerell AD, Nilsson L, Petrella RJ (2009). CHARMM: the biomolecular simulation program.. J Comput Chem.

[49] Montes M, Miteva MA, Villoutreix BO (2007). Structure-based virtual ligand screening with LigandFit: pose prediction and enrichment of compound collections.. Proteins.

[50] Ganesan A (2008). The impact of natural products upon modern drug discovery.. Curr Opin Chem Biol.

[51] Keller TH, Pichota A, Yin Z (2006). A practical view of ‘druggability’.. Curr Opin Chem Biol.

[52] Khan MT (2010). Predictions of the ADMET properties of candidate drug molecules utilizing different QSAR/QSPR modelling approaches.. Curr Drug Metab.

[53] Wallace AC, Laskowski RA, Thornton JM (1995). LIGPLOT: a program to generate schematic diagrams of protein-ligand interactions.. Protein Eng.

[54] Noronha G, Barrett K, Boccia A, Brodhag T, Cao JG (2007). Discovery of [7-(2,6-dichlorophenyl)-5-methylbenzo [1,2,4]triazin-3-yl]-[4-(2-pyrrolidin-1-ylethoxy)phenyl]amine - a potent, orally active Src kinase inhibitor with anti-tumor activity in preclinical assays.. Bioorg Med Chem Lett.

[55] Kowar TR (1998). Genetic function approximation experimental design (GFAXD): A new method for experimental design.. J Chem Inf Comp Sci.

[56] Jamois EA (2004). Analysis of multiple QSAR models from genetic function approximation: A basis for experimental design.. Abstr Pap Am Chem S.

[57] Slinker BK, Glantz SA (2008). Multiple linear regression: accounting for multiple simultaneous determinants of a continuous dependent variable.. Circulation.

[58] MATrix LABoratory.

[59] Hasegawa K, Funatsu K (2010). Non-linear modeling and chemical interpretation with aid of support vector machine and regression.. Curr Comput Aided Drug Des.

[60] Koh IS, Kim JH, Lee J, Oh B, Kimm K (2004). Prediction of phosphorylation sites using SVMs.. Bioinformatics.

[61] Chang C-C, Lin C-J (2011). LIBSVM: A Library for Support Vector Machines.. ACM Transcations on Intelligent Systems and Technology.

[62] SYBYL-X. 1.1 ed.

