# Supplementary material for: In Silico Investigation of Potential Src Kinase Ligands from Traditional Chinese Medicine
Source: PLoS One. 2012 Mar 21;7(3):e33728. doi: 10.1371/journal.pone.0033728 (PMC3312348; doi:10.1371/journal.pone.0033728)
Supplement: Table S1 — Predicted and observed activity (pIC50) of 53 known Src kinase inhibitors1 using generated CoMFA and CoMSIA models. (DOC) [file pone.0033728.s001.doc]

Table S1.

| Compound | Observed | CoMFA | | CoMSIA | |
| --- | --- | --- | --- | --- | --- |
|  |  | Predicted | Residual | Predicted | Residual |
| 1 | 8.155 | 7.950 | 0.205 | 7.814 | 0.341 |
| 2 | 6.467 | 6.237 | 0.230 | 6.367 | 0.100 |
| 3* | 7.959 | 6.893 | 1.066 | 7.138 | 0.821 |
| 4* | 7.456 | 7.014 | 0.442 | 6.877 | 0.579 |
| 5 | 6.682 | 6.849 | -0.167 | 6.641 | 0.041 |
| 6 | 6.297 | 6.759 | -0.462 | 6.563 | -0.266 |
| 7 | 6.109 | 6.079 | 0.030 | 6.407 | -0.298 |
| 8 | 5.921 | 5.822 | 0.099 | 6.010 | -0.089 |
| 9 | 6.690 | 6.492 | 0.198 | 6.784 | -0.094 |
| 10 | 6.684 | 6.737 | -0.053 | 6.592 | 0.092 |
| 11 | 5.824 | 6.080 | -0.256 | 5.866 | -0.042 |
| 12 | 5.745 | 5.974 | -0.229 | 6.114 | -0.369 |
| 13 | 7.824 | 7.677 | 0.147 | 7.714 | 0.110 |
| 14 | 7.523 | 7.620 | -0.097 | 7.468 | 0.055 |
| 15 | 5.000 | 4.626 | 0.374 | 5.006 | -0.006 |
| 16* | 7.921 | 7.787 | 0.134 | 7.927 | -0.006 |
| 17* | 8.046 | 7.282 | 0.764 | 7.886 | 0.160 |
| 18 | 7.854 | 6.945 | 0.909 | 7.168 | 0.686 |
| 19 | 7.886 | 7.802 | 0.084 | 8.011 | -0.125 |
| 20* | 7.602 | 7.026 | 0.576 | 7.070 | 0.532 |
| 21 | 7.292 | 7.146 | 0.146 | 7.362 | -0.070 |
| 22 | 6.550 | 6.754 | -0.204 | 6.619 | -0.069 |
| 23* | 6.452 | 6.647 | -0.195 | 6.497 | -0.045 |
| 24 | 6.200 | 6.398 | -0.198 | 6.065 | 0.135 |
| 25 | 5.538 | 5.521 | 0.017 | 5.408 | 0.130 |
| 26 | 7.959 | 7.996 | -0.037 | 8.145 | -0.186 |
| 27 | 6.502 | 6.601 | -0.099 | 6.845 | -0.343 |
| 28 | 5.000 | 5.667 | -0.667 | 5.280 | -0.280 |
| 29 | 5.000 | 5.420 | -0.420 | 4.554 | 0.446 |
| 30 | 8.000 | 7.652 | 0.348 | 7.410 | 0.590 |
| 31 | 7.174 | 7.344 | -0.170 | 7.674 | -0.500 |
| 32 | 7.921 | 8.020 | -0.099 | 7.861 | 0.060 |
| 33 | 7.699 | 7.780 | -0.081 | 7.566 | 0.133 |
| 34 | 7.036 | 5.997 | 1.039 | 7.184 | -0.148 |
| 35 | 7.143 | 6.953 | 0.190 | 6.897 | 0.246 |
| 36 | 8.000 | 8.029 | -0.029 | 7.971 | 0.029 |
| 37* | 8.000 | 7.338 | 0.662 | 7.534 | 0.466 |
| 38 | 7.481 | 7.375 | 0.106 | 7.428 | 0.053 |
| 39 | 7.886 | 7.871 | 0.015 | 7.652 | 0.234 |
| 40 | 8.194 | 8.048 | 0.146 | 8.165 | 0.029 |
| 41* | 7.678 | 7.479 | 0.199 | 7.288 | 0.390 |
| 42 | 7.398 | 7.424 | -0.026 | 7.594 | -0.196 |
| 43 | 7.444 | 7.447 | -0.003 | 7.564 | -0.120 |
| 44 | 7.409 | 7.224 | 0.185 | 7.376 | 0.033 |
| 45 | 7.824 | 7.891 | -0.067 | 7.915 | -0.091 |
| 46 | 7.886 | 7.860 | 0.026 | 7.946 | -0.060 |
| 47 | 7.796 | 7.746 | 0.050 | 7.854 | -0.058 |
| 48* | 7.745 | 7.839 | -0.094 | 7.907 | -0.162 |
| 49 | 8.194 | 8.493 | -0.299 | 7.964 | 0.230 |
| 50 | 8.000 | 7.877 | 0.123 | 7.913 | 0.087 |
| 51 | 8.000 | 7.811 | 0.189 | 7.497 | 0.503 |
| 52* | 7.854 | 7.619 | 0.235 | 7.426 | 0.428 |
| 53 | 7.678 | 8.03 | -0.352 | 7.869 | -0.191 |

^1^: Observed inhibitory activities adapted from .

Comp.: compounds

Obs.: observed

Pre.:predicted

*: test set
